# Assessing the Impact of Precision Parameter Prior in Bayesian Non-parametric Growth Curve Modeling

**DOI:** 10.3389/fpsyg.2021.624588

**Published:** 2021-03-31

**Authors:** Xin Tong, Zijun Ke

**Affiliations:** ^1^Department of Psychology, University of Virginia, Charlottesville, VA, United States; ^2^Department of Psychology, Sun Yat-sen University, Guangzhou, China

**Keywords:** non-parametric Bayesian, robust method, growth curve modeling, Dirichlet process mixture, prior, precision parameter

## Abstract

Bayesian non-parametric (BNP) modeling has been developed and proven to be a powerful tool to analyze messy data with complex structures. Despite the increasing popularity of BNP modeling, it also faces challenges. One challenge is the estimation of the precision parameter in the Dirichlet process mixtures. In this study, we focus on a BNP growth curve model and investigate how non-informative prior, weakly informative prior, accurate informative prior, and inaccurate informative prior affect the model convergence, parameter estimation, and computation time. A simulation study has been conducted. We conclude that the non-informative prior for the precision parameter is less preferred because it yields a much lower convergence rate, and growth curve parameter estimates are not sensitive to informative priors.

## 1. Introduction

Bayesian non-parametric (BNP) modeling, also called semiparametric Bayesian modeling in the literature, has been recognized as a valuable data analytical technique due to its great flexibility and adaptivity (e.g., Müller and Mitra, 2004; Gershman and Blei, 2012). It is rapidly gaining popularity among methodologists and practitioners and has been applied to a variety of models including regressions, latent variable models with complex structures, sequential models, etc. BNP models are on an infinite dimensional parameter space and the complexity of the models adapts to the data. One of the most popular BNP models is Dirichlet process (DP) mixtures. Being able to adapt the number of latent classes to the complexity of the data, DP mixtures are powerful in modeling empirical data. However, they also face technical challenges. One challenge is the estimation of the precision parameter in the DP mixture. In this study, we focus on the prior of precision parameter and investigate how it affects model convergence, parameter estimation, and computation time in BNP growth curve modeling.

Growth curve models are broadly used in longitudinal research (e.g., Meredith and Tisak, 1990; McArdle and Nesselroade, 2014). Many popular longitudinal models in social and behavioral sciences, such as multilevel models, some mixed-effects models, and linear hierarchical models, can be written as a form of growth curve models. In growth curve models, dependent variables are repeatedly measured and explained as a function of time and possible control variables. The mean function between the dependent variables and time is the mean growth. Random effects and measurement errors cause the individual growth trajectories to deviate from the mean growth curve. Traditional growth curve modeling is typically based on the normality assumption. That is, both the random effects and measurement errors are assumed to follow normal distributions. However, empirical data often violate the normality assumption (Micceri, 1989; Cain et al., 2017). Non-normal population distributions and data contamination are two common causes of non-normality. Although standard errors and test statistics have been corrected to reduce the adverse effect of distributional assumption violation (e.g., Chou et al., 1991; Curran et al., 1996), normal-distribution-based maximum likelihood estimation may still yield inefficient or inaccurate parameter estimates, and thus misleading statistical inferences (e.g., Yuan and Bentler, 2001; Maronna et al., 2006). Therefore, researchers have developed robust methods to obtain accurate parameter estimation and statistical inference.

The ideas of robust methods can be divided into two types. For the first type, the key idea is to downweight extreme cases. To do so, this type of robust methods assigns a weight to each subject in a dataset according to its distance from the center of the majority of the data (e.g., Pendergast and Broffitt, 1985; Singer and Sen, 1986; Silvapulle, 1992; Yuan and Bentler, 1998; Zhong and Yuan, 2010). For the second type, the key idea is to use non-normal distributions that are mathematically tractable while building the statistical model. For example, latent variables and/or measurement errors are assumed to follow a *t* or skew-*t* distribution (Tong and Zhang, 2012; Zhang, 2016) or a mixture of certain distributions (Muthén and Shedden, 1999; Lu and Zhang, 2014). While being useful, these methods have limitations under certain conditions. For example, the downweighting method does not perform well when latent variables contain extreme scores (see Zhong and Yuan, 2011). Using a *t* distribution or a mixture of normal distributions still imposes restrictions on the shape of the data distribution.

The aforementioned issues are automatically resolved by BNP methods. BNP modeling relies on a building block, DP, to handle the non-normality issue. DP is a distribution over probability measures that can be used to estimate unknown distributions. Consequently, the non-normality issue can be addressed by directly estimating the unknown random distributions of latent variables or measurement errors (i.e., obtaining the posteriors of the distributions).

The advantages of using BNP methods with DP priors have been discussed in the literature (e.g., Ghosal et al., 1999; MacEachern, 1999; Hjort, 2003; Müller and Mitra, 2004; Fahrmeir and Raach, 2007; Hjort et al., 2010). They do not constrain models to a specific parametric form which may limit the scope and type of statistical inferences in many situations, especially when data are not normally distributed. Thus, a typical motivation of using BNP methods is that one is unwilling to make somewhat arbitrary and unverified assumptions for latent variables or error distributions as in the parametric modeling. Meanwhile, BNP methods can provide full probability models for the data-generating process and lead to analytically tractable posterior distributions.

BNP methods have been applied to complex models. For example, Bush and MacEachern (1996), Kleinman and Ibrahim (1998), and Brown and Ibrahim (2003) used DP mixtures to handle non-normal random effects. Burr and Doss (2005) used a conditional DP to handle heterogeneous effect sizes in the context of meta-analysis. Ansari and Iyengar (2006) included Dirichlet components to build a semiparametric recurrent choice model. Dunson (2006) used dynamic mixtures of DP to estimate the varied distributions of a latent variable, which change non-parametrically across groups. Si and Reiter (2013), Si et al. (2015) used DP mixtures of multinomial distributions for categorical data with missing values. BNP approach has also been adapted to structural equation modeling to relax the normality assumption of the latent variables (e.g., Lee et al., 2008; Yang and Dunson, 2010). Tong and Zhang (2019) directly used a DP mixture to model non-normal data in growth curve modeling.

Although the application of BNP modeling has increased dramatically since the theoretical properties of BNP methods were better understood and their computational hurdles were removed (e.g., Neal, 2000), BNP modeling is still unfamiliar to the majority of researchers in social and behavioral sciences. Additionally, there are technical issues that have not yet been fully addressed (Sharif-Razavian and Zollmann, 2009). The convergence issue is one of such unanswered questions. Non-convergence can occur when BNP method is applied to complex models. Tong and Zhang (2019) found that non-convergence was largely caused by the precision parameter of the mixing DP. The precision parameter is a critical hyperparameter that governs the expected number of mixture components. When a non-informative prior was used for the precision parameter, non-convergence occurred or a longer computation time was observed (Tong and Zhang, 2019). Informative priors may help solve this issue. However, only a few studies have noticed and discussed the effect of the precision parameter in DP mixtures (e.g., West, 1992; Ohlssen et al., 2007; Jara et al., 2011). Ishwaran (2000) was among the few that studied the informative prior for the precision parameter. Ishwaran (2000) suggested to use the *Gamma*(2, 2) prior to encourage both small and large values of the precision parameter. In sum, despite its impact on the model convergence issue, no study has systematically investigated how the prior for the precision parameter should be specified.

Therefore, in this study, we evaluate and compare non-informative, weakly informative, accurate informative, and inaccurate informative priors for the precision parameter of DP mixtures. We study how these priors influence model convergence, model estimation, and computation time in BNP growth curve modeling. In the next section, we introduce BNP growth curve modeling. After providing the conditional posterior distribution of the precision parameter, we use a simulation study to assess the impact of four types of priors for the precision parameter. Recommendations are provided at the end of the article. We also provide a guideline about the implementation of BNP growth curve modeling using R (R Core Team, 2019) in the Appendix.

## 2. Bayesian Non-parametric Growth Curve Modeling

We now introduce a typical growth curve model and a BNP method based on this model. Consider a longitudinal dataset with *N* subjects and *T* measurement occasions. Let ${\text{y}}_{i}={\left(y_{i1},...,y_{iT}\right)}^{\prime }$ be a *T* × 1 random vector with *y*_*ij*_ being a measurement from individual *i* at time *j* (*i* = 1, . . . , *N*; *j* = 1, . . . , *T*). A growth curve model without covariates can be written as

$$\begin{array}{l} y_{i}=\Lambda b_{i}+e_{i},\\ b_{i}=\beta +u_{i}, \end{array}$$

where **Λ** is a *T* × *q* factor loading matrix that determines the growth curves, **b**_*i*_ is a *q* × 1 vector of random effects, and **e**_*i*_ is a vector of measurement errors. The vector of random effects **b**_*i*_ varies around its mean ***β***. The residual vector **u**_*i*_ represents the deviation of **b**_*i*_ from ***β***. When

$$\text{Λ}=\left(\begin{array}{cc} 1 & \;0\\ 1 & \;1\\ \vdots & \;\vdots \\ 1 & \;T-1 \end{array}\right)\;,b_{i}=\left(\begin{array}{c} L_{i}\\ S_{i} \end{array}\right)\;,\;and\;\beta =\left(\begin{array}{c} \beta_{L}\\ \beta_{S} \end{array}\right)\;,$$

the model is reduced to a linear growth curve model with random intercept *L*_*i*_ and random slope *S*_*i*_. The mean intercept and slope are denoted as *β*_*L*_ and *β*_*S*_, respectively.

Traditionally, **e**_*i*_ and **u**_*i*_ are assumed to follow multivariate normal distributions with mean vectors of zero and covariance matrices **Φ** and **Ψ**, respectively, so **e**_*i*_ ~ *MN*_*T*_(**0**, **Φ**) and **u**_*i*_ ~ *MN*_*q*_(**0**, **Ψ**). Here, *MN* denotes a multivariate normal distribution and its subscript indicates its dimension. Measurement errors are often assumed to be uncorrelated with each other and have equal variances across time. Statistically, this simplification means the covariance matrix of measurement error **Φ** is reduced to $\Phi =\sigma_{e}^{2}\text{I}$ where $\sigma_{e}^{2}$ is a scale parameter. In linear growth curve models, ${\text{u}}_{i}={\left(u_{Li},u_{Si}\right)}^{\prime }$. Its covariance matrix is then $\text{Ψ}=cov\left({\text{u}}_{i}\right)=\left(\begin{array}{cc} \sigma_{L}^{2} & \;\sigma_{LS}\\ \sigma_{LS} & \;\sigma_{S}^{2} \end{array}\right)$. Here, $\sigma_{L}^{2}$ and $\sigma_{S}^{2}$ represent the variances of the random intercept and slope across individuals, respectively, and *σ*_*LS*_ represents the covariance between the random intercept and slope.

BNP methods do not make arbitrary distributional assumptions as in the parametric modeling and thus are more flexible in handling non-normal data (e.g., Lee et al., 2008; Tong and Zhang, 2019). Unlike conventional non-parametric methods such as permutation tests, BNP methods use full probability models to describe the data-generating process and thus can derive posterior distributions for model parameters.

Within the BNP modeling scope, the parametric distributions of latent variables and measurement errors in traditional methods are replaced by unknown random distributions. To estimate these unknown distributions, DP is frequently used as the prior (Ferguson, 1973, 1974). Specifically, a random “sample” from a DP is a random distribution. Here, we denote it as *G*. A DP has two hyperparameters, *α* and *G*_0_. The base distribution, *G*_0_, represents the central tendency or “mean” distribution in the distribution space. The precision parameter, *α*, quantifies how far away realizations of *G* deviate from *G*_0_. According to Ferguson (1973), DP is a conjugate prior that has two desirable properties: (1) a sufficiently large support, and (2) analytically manageable posterior distributions. Ferguson further derived the posterior of *G*, $DP\left(\tilde{\alpha },\tilde{G_{0}}\right)$. Here, $\tilde{\alpha }=\alpha +N$ and

$$\tilde{G_{0}}=\frac{\alpha }{\alpha +N}G_{0}+\frac{N}{\alpha +N}G_{N}$$

with *G*_*N*_ being the empirical distribution of the data. Notably, the posterior point estimate of *G*, $E\left(G|data\right)=\tilde{G_{0}}$, is a weighted average of the base distribution or prior mean *G*_0_ and the empirical distribution or data *G*_*N*_. When *α* = 0, the posterior point estimate is reduced to the empirical distribution *G*_*N*_, which is pure non-parametric. When *α* approaches to infinity, the posterior point estimate gradually approximates *G*_0_, which is parametric. A common practice is to specify a gamma prior for *α*, which would yield a posterior estimate that is neither 0 nor infinity.

In BNP growth curve modeling, latent variables and/or measurement errors can be modeled non-parametrically. In this article, we focus on the distributional assumption of measurement errors. When the normality of measurement errors is suspected, we assume that **e**_*i*_ ~ *G*_*e*_ where *G*_*e*_ is an unknown random distribution that is determined by the data. In the BNP framework, DP is typically adopted to specify *G*_*e*_. Because the distribution of **e**_*i*_ is continuous but DP is essentially discrete, a DP mixture (DPM) can be used to model the measurement errors such that

$$G_{e}=\{\begin{array}{ll} D(\mu_{e}^{\left(1\right)},\Phi^{\left(1\right)}), & with\;p=p_{1}\\ D(\mu_{e}^{\left(2\right)},\Phi^{\left(2\right)}), & with\;p=p_{2}\\ \vdots & \vdots \\ D(\mu_{e}^{\left(k\right)},\Phi^{\left(k\right)}), & with\;p=p_{k}\\ \vdots & \vdots \end{array},$$

where *D* represents a predetermined multivariate distribution (e.g., multivariate normal, *t*, multinomial, etc.), and $\mu_{e}^{\left(k\right)}\;\text{and}\;\Phi^{\left(k\right)},k=1,...,\infty$ are means and covariances of the multivariate distribution in the *k*th component with probability *p*_*k*_. Theoretically, given an arbitrary distributional shape, there could be infinite number of mixture components as *k* goes to infinity. In practice, a finite number of mixture components often can describe a distribution well and the number of mixture components is determined by the DP precision parameter *α*. Smaller *α* yields a smaller number of mixture components. If *α* approaches infinity, there would be *N* mixture components, one associated with each subject. Namely, the precision parameter *α* is an important parameter that can determine the complexity of the model and how well the model fits the data, and thus may affect the convergence of the model. For the intraindividual measurement errors in the typical linear growth curve model, Tong and Zhang (2019) proposed that

$$\begin{array}{l} e_{i}|\Phi_{i}\sim MN_{T}(0,\Phi_{i}),\\ \Phi_{i}|G\;\sim G,\\ \;G\sim DP(\alpha ,G_{0}). \end{array}$$

That is, the unknown distribution *G*_*e*_ is approximated by a mixture of multivariate normal distributions where the mixing measure has a DP prior, *G*_*e*_ ~ *DPM*. The DP prior *DP*(*α*, *G*_0_) can be obtained using the truncated stick-breaking construction (e.g., Sethuraman, 1994; Lunn et al., 2013). Specifically, $DP\left(\cdot \right)=\overset{C}{\underset{j=1}{\sum }}p_{j}\delta_{z_{j}}\left(\cdot \right),1\leq C<\infty$, where *C* (1 ≤ *C* ≤ *N*, often set at a large number) is a possible maximum number of mixture components, δ_*z*_*j*__(·) denotes a point mass at *z*_*j*_ and *z*_*j*_ ~ *G*_0_ independently. The random weights *p*_*j*_ can be generated through the following procedure. With *q*_1_, *q*_2_, . . . , *q*_*C*_ ~ *Beta*(1, *α*), define

$$p_{j}^{'}=q_{j}\prod_{k=1}^{j-1}\left(1-q_{k}\right),j=1,...,C.$$

Then, *p*_*j*_ is obtained by

(1) $$p_{j}=\frac{p_{j}^{'}}{\sum_{K=1}^{C}p_{k}^{'}},$$

to satisfy that $\overset{C}{\underset{j=1}{\sum }}p_{j}=1$. In practice, the updating of **e**_*i*_ can proceed as in a typical DP mixture model and its distribution is an infinite mixture distribution^1^.

In general, the distribution of **e**_*i*_ through the truncated stick-breaking construction is

$$G_{e}=\{\begin{array}{ll} D(\mu_{e}^{\left(1\right)},\Phi^{\left(1\right)}), & with\;p=p_{1}\\ D(\mu_{e}^{\left(2\right)},\Phi^{\left(2\right)}), & with\;p=p_{2}\\ \vdots & \vdots \\ D(\mu_{e}^{\left(C\right)},\Phi^{\left(C\right)}), & with\;p=p_{C} \end{array},$$

where *D* represents a predetermined multivariate distribution, $\mu_{e}^{\left(j\right)}\;\text{and}\;\Phi^{\left(j\right)},j=1,...,C$ are means and covariances of the multivariate distribution in the *j*th component, and *p*_*j*_ is obtained using Equation (1). Given that the mean of **e**_*i*_ is **0**, we constrain $\overset{C}{\underset{j=1}{\sum }}p_{j}\mu_{e}^{\left(j\right)}=\text{0}$. For simplicity, in this study, we follow Tong and Zhang (2019) and use multivariate normal distributions for the mixing components and constrain $\mu_{e}^{\left(j\right)}$ to be 0. We use inverse Wishart priors $p\left(\Phi^{\left(j\right)}\right)=IW\left(n_{0},W_{0}\right)$ for the covariance matrices of the mixture components, **Φ**^(*j*)^, *j* = 1, . . . , *C*. Following Lunn et al. (2013, p. 294), we fix the shape parameter *n*_0_ at a specific number and assign an inverse Wishart prior to the scale matrix *W*_0_. With such a specification, the measurement error for individual *i*, **e**_*i*_, has a *p*_*j*_ probability of coming from the mixing component *MN*(**0**, **Φ**^(*j*)^). The measurement errors for other individuals may also come from the same mixing component. Let *K* denotes the number of mixing components or *MN*(**0**, **Φ**^(*j*)^) with *j* = 1, . . . , *C*. In other words, *K* is the number of latent classes for **e**_*i*_ and *K* can be smaller than *C*, *K* ≤ *C*. Within each class, **e**_*i*_s come from the same distribution.

We would like to note that a similar approach to BNP modeling is finite mixture modeling (FMM). FMM estimates or equivalently approximates an unknown distribution using a mixture of known distributions. A key difference between FMM and BNP modeling is that the number of mixture components is treated as known in FMM, whereas this number is treated as unknown and is freely estimated in BNP modeling. As a result, when FMM is used to handle non-normality, additional analyses such as model comparison are needed to determine the unknown number of mixture components. BNP modeling therefore is believed to have the advantage of being more objective and data-driven, given that additional analyses such as model comparison that may be vulnerable to subjectivity are avoided.

Bayesian methods are applied to estimate BNP growth curve models. Bayesian methods are becoming increasingly popular in recent years because of their flexibility and powerfulness in estimating models with complex structures (e.g., Lee and Shi, 2000; Lee and Song, 2004; Zhang et al., 2007; Lee and Xia, 2008; Tong and Zhang, 2012; Serang et al., 2015). The key idea of Bayesian methods is to compute the posterior distributions for model parameters by combining the likelihood function and the priors. As introduced previously, ***β***, **Φ**, and **Ψ** are the model parameters in traditional growth curve model. In a BNP growth curve model, ***β*** and **Ψ** remain model parameters. In contrast, the measurement error covariance matrix **Ψ** is not directly estimated. Instead, we obtain **e**_*i*_ based on which we can get **Φ**. Another important parameter in BNP growth curve modeling is the precision parameter *α*. Let *p*(***β***, **Ψ**, *α*) be the joint prior distribution of model parameters, and let *L* be the likelihood function. The joint posterior distribution of model parameters is

$$p(\beta ,\Psi ,\alpha |y_{i})\propto \int p(\beta ,\Psi ,\alpha )\times L\;db,$$

where $b={\left(b_{1}^{'},\;...,b_{N}^{'}\right)}^{'}.$. It is difficult to solve for this integral in practice. Instead, Markov chain Monte Carlo (MCMC) methods (e.g., Gibbs sampling; Robert and Casella, 2004) are often used to obtain parameter estimates and statistical inferences. Specifically, we first derive the conditional posterior distribution for each of the parameters. We then iteratively draw samples from the derived conditional posteriors to obtain empirical marginal distributions of the model parameters. Finally, statistical inferences are made based on the empirical marginal distributions (Geman and Geman, 1984).

## 3. Precision Parameter in BNP Models

The convergence issue in BNP growth curve modeling is likely related to the precision parameter (Tong and Zhang, 2019). Here, we provide a theoretical discussion on how the prior of the precision parameter can influence the number of latent classes for **e**_*i*_.

The DP precision parameter *α* is the key to govern the expected number of latent classes. It directly determines the distribution of *K*, the number of latent classes of **e**_*i*_. With a larger *K*, measurement errors of different individuals are more likely to have different distributions. West (1992) found that *K* asymptotically follows a Poisson distribution

(2) $$\begin{array}{r} K=1+x,\;x\sim Poisson\left(\alpha \left(\gamma +logN\right)\right) \end{array}$$

where *γ* is Euler's constant. Several percentiles of the distribution of *K* are given in Table 1. As shown in the table, *K* increases as *α* and *N* increases.

**Table 1:** Different percentiles (5, 50, 95%) of the distribution of the number of clusters *K*, given different values of precision parameter *α*, and sample size *N*.

	***α*** = ***0.1***			***α*** = ***1***			***α*** = ***2***		
	**5%**	**50%**	**95%**	**5%**	**50%**	**95%**	**5%**	**50%**	**95%**
*N* = 200	1	1	3	3	7	11	7	13	19
*N* = 600	1	2	3	4	8	13	9	15	21
*N* = 1,000	1	2	3	4	8	13	10	16	23

As discussed previously, a gamma prior *Gamma*(*a*_1_, *a*_2_) is often used for the hyperparameter *α*. Given such a prior, West (1992) derived the posterior of *α* as a mixture of two gamma densities

$$\begin{array}{l} \alpha |\text{· }\sim \pi_{x}Gamma(a_{1}+K,a_{2}-logx)\\ \;+(1-\pi_{x})Gamma(a_{1}+K-1,a_{2}-logx), \end{array}$$

where *x* is an augmented variable *x*|· ~ *Beta*(*α* + 1, *N*) and the weights π_*x*_ is defined by $\pi_{x}/\left(1-\pi_{x}\right)=\frac{a_{1}+K-1}{N\left(a_{2}-logx\right)}$. Although West (1992) also provided an approximation to the posterior of *α*, *p*(*α*|·) ≈ *Gamma*(*a*_1_ + *K* − 1, *a*_2_ + *γ* + *logN*), how good the approximation was has not been investigated.

A non-informative prior for *α* seems to be reasonable, especially when the information about number of latent classes are not available. However, a non-informative prior may cause non-convergence of Markov chains. Therefore, it is worth evaluating different priors for the precision parameter.

## 4. A Simulation Study

We now present a simulation study to evaluate the influence of the prior for the precision parameter in BNP growth curve modeling when data are normally distributed and contain outliers^2^. The linear growth curve model in the previous section is used. Measurement errors are modeled non-parametrically to address the non-normality. Based on the results of previous studies, the number of times points (*T*), the covariance between the random intercept and slope (*σ*_*LS*_), and the measurement error variance ($\sigma_{e}^{2}$) have trivial effects on the performance of BNP growth curve modeling (e.g., Tong and Zhang, 2019). Therefore, we only consider a set of values for these parameters in this study. We follow the empirical data analysis results in Tong and Zhang (2019) to select the population parameter values: the fixed effects are fixed at $\beta ={\left(\beta_{L},\beta_{S}\right)}^{\prime }={\left(6.2,0.3\right)}^{\prime }$; the number of measurement occasion is *T* = 4; measurement error variance $\sigma_{e}^{2}=0.5$; variances of the random intercept and slope are 1 and 0.1, respectively, and the covariance between the random intercept and slope *σ*_*LS*_ = 0.

**Figure 1:**
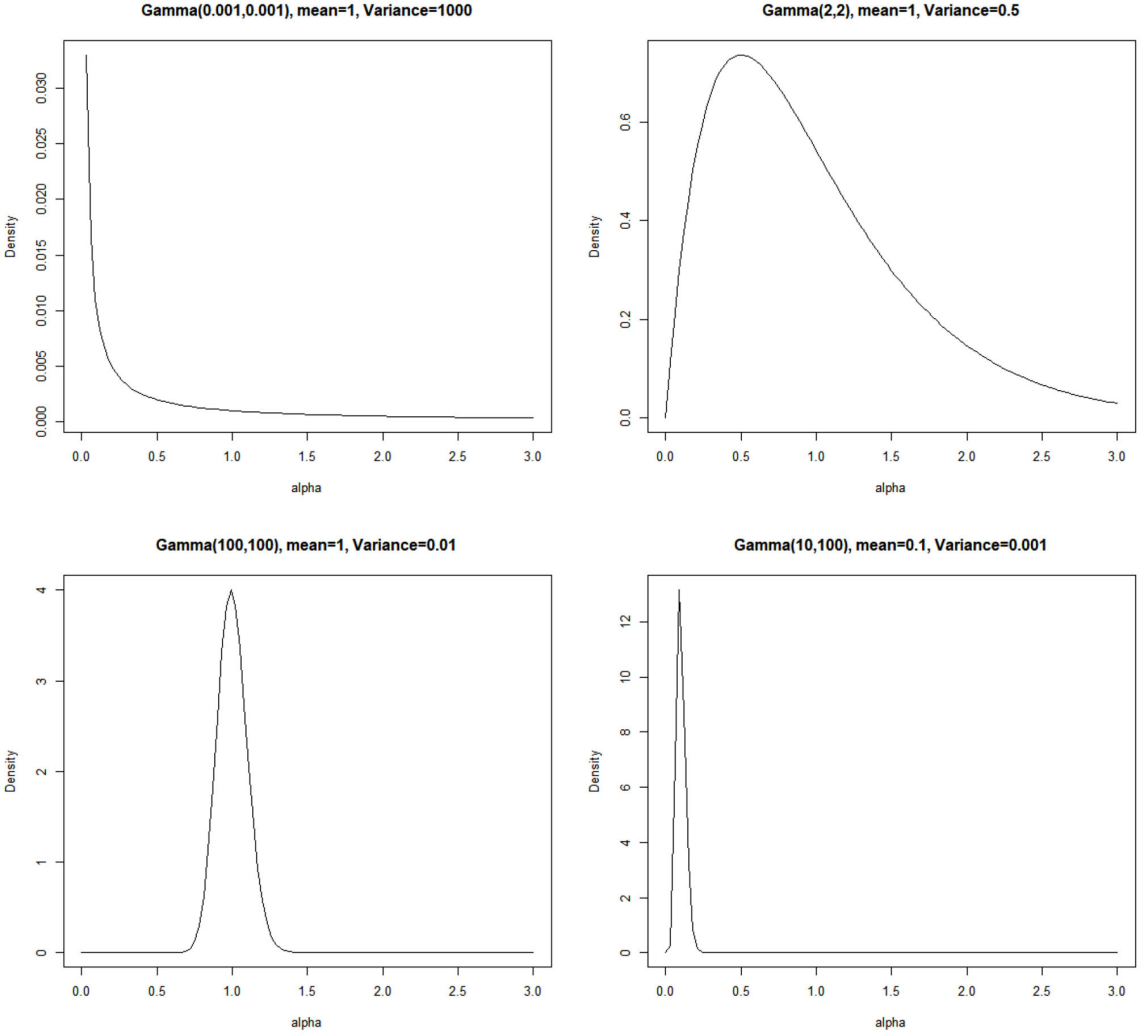
Density curves for the four precision parameter priors used in the simulation study.

Three potentially influential factors are manipulated in the simulation study, including sample size, data distribution, and precision parameter prior. First, two sample sizes are considered, *N* =200 or 600, representing small and large sample sizes. Second, data are either normal or containing outliers. When generating outliers, three proportions of outliers are considered, *r%* =5, 10, or 20%. To generate outliers, we randomly select *r%* observations at each measurement occasion and replace them by extreme values. The extreme values are generated from 10 different distributions with a large mean of *L*_*i*_ + *S*_*i*_(*j* − 1) +* m*σ**_*e*_ where *m* ≥ 5 is generated from a truncated Poisson distribution, and a variance of $\sigma_{e}^{2}$ which is the same as that of the normal data. As a result, the true distribution of the data is a mixture of 11 distributions. Outliers generated in this way conform to the definition of outliers (Yuan and Zhong, 2008; Tong and Zhang, 2017). See Supplementary Figures 1, 2 to aid the understanding of the shape of generated normal data and data with outliers. Third, four priors for the precision parameter are investigated (see Figure 1): a diffuse prior *Gamma*(0.001, 0.001), a weakly informative prior *Gamma*(2, 2) suggested by Ishwaran (2000), an accurate informative prior *Gamma*(100, 100), and an inaccurate informative prior *Gamma*(10, 100). Gamma(10,100) is an inaccurate informative prior because its mean is 0.1 and its variance is as small as 0.001. According to Table 1, the resulting number of latent classes ranges from 1 to 3, whereas the true number of mixed underlying distribution is 11. For all the other model parameters, conventional non-informative priors such as those in Zhang et al. (2013) are used. Specifically, fixed effects *β* have non-informative diffuse priors *N*(0, 10^6^). The covariance matrix of the random intercept and slope **Ψ** has an inverse-Wishart prior with an identity scale matrix and degrees of freedom being 2.

In each simulation condition, 500 datasets are generated. BNP growth curve modeling is applied for each dataset using JAGS with the rjags package in software R (Plummer, 2017; R Core Team, 2019). The total length of Markov chains is set at 50,000 and the first half of iterations is the burn-in period^3^. We assess how different priors affect model convergence rate, parameter estimation, and computation time.

Geweke tests (Geweke, 1991) are used to perform the convergence diagnostics. After the burn-in period, if parameter values are sampled from the stationary distribution of the chain, the means of the first and last parts of the Markov chain (by default the first 10% and the last 50%) should be equal and Geweke's statistic asymptotically follows a standard normal distribution. A Markov chain converges when the Geweke's statistic is between –1.96 and 1.96. If none of the convergence diagnostics (i.e., Geweke tests) for all model parameters suggest non-convergence, the model is said to have converged. In each simulation condition, the convergence rate is defined as the proportion of converged models out of the total 500 generated replications.

For the assessment of model estimation, we obtain the parameter estimate bias, average standard error (ASE), empirical standard error (ESE), mean squared error (MSE), and coverage probability (CP) of the 95% highest posterior density (HPD) credible intervals for each parameter based on converged simulation replications^4^.

In addition, the estimation time (in seconds) is recorded for each replication. The average estimation time (AET) is the average of the estimation time for all the converged replications.

All program code and detailed results for the simulation study are available on our GitHub site: https://github.com/CynthiaXinTong/PrecisionParPrior_BNP_GCM.

### 4.1. Main Results

**Figure 2:**
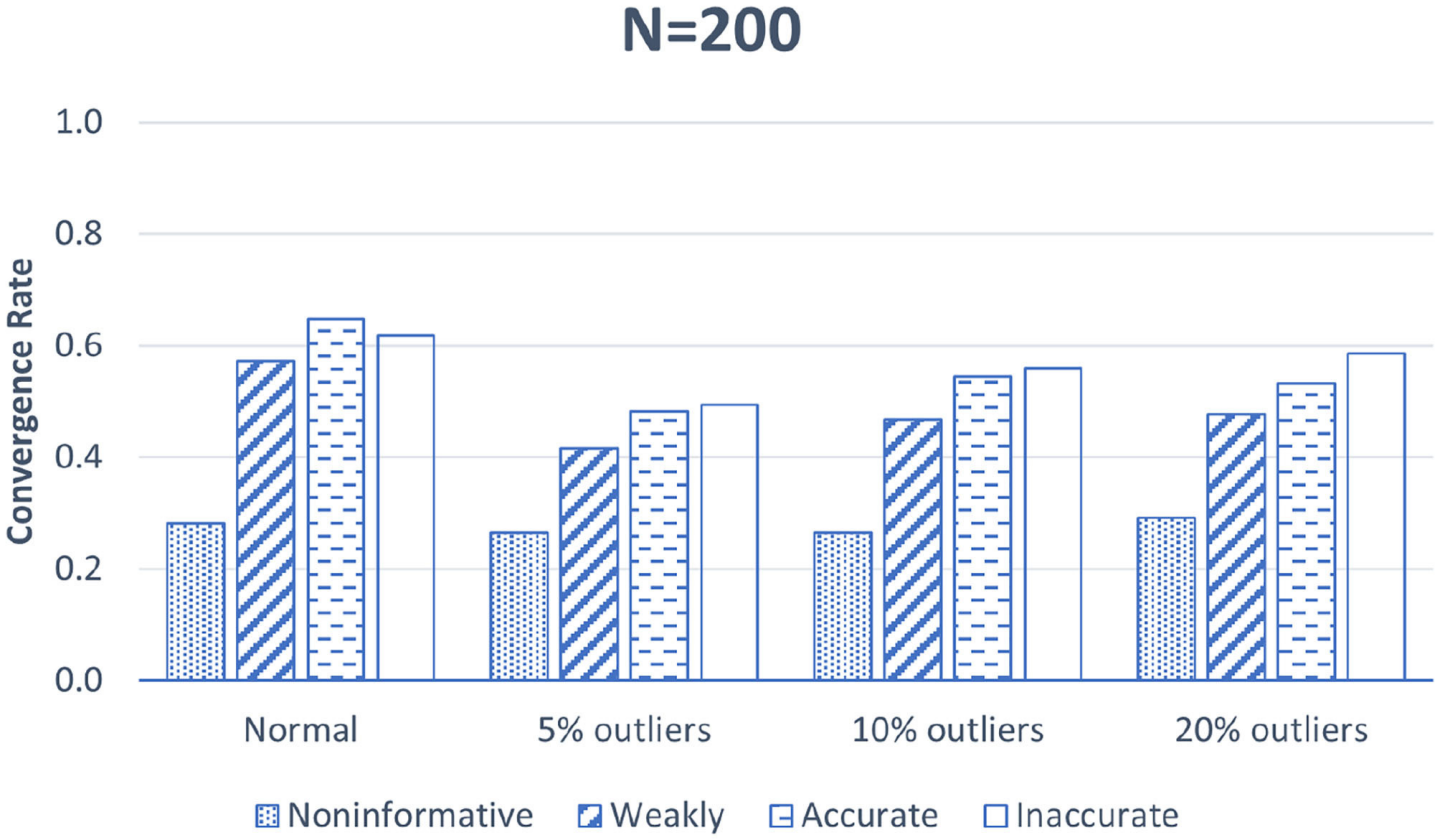
Convergence rate for different priors when *N* = 200.

Figure 2 shows the convergence rate for BNP growth curve modeling with different precision parameter priors when sample size is 200. This figure clearly shows that outliers harm model convergence. Note that the convergence rate for data with 5% outliers is the lowest. This may be because a small proportion of outliers (e.g., 5%) creates a steep and high-curvature region for the Markov chain to enter and thus more difficult to converge. As the outlier proportion increases, the curvature becomes smoother so the convergence rate is higher. Among the four studied priors, the non-informative prior for the precision parameter always leads to the lowest convergence rate, i.e., less than 30% across all the simulation conditions. Informative priors substantially increase the model convergence rate. Specifically, the convergence rate doubles when we switch from the non-informative prior to the weakly informative prior suggested by Ishwaran (2000) in the condition with normal data. The incremental amount is about 30% of the original convergence rate in the conditions with outliers. Both accurate informative priors and inaccurate informative priors lead to higher convergence rates. The importance of using informative priors is more salient when data are not normal. Note that inaccurate informative priors yield slightly higher convergence rates than accurate informative priors because the variance of the inaccurate prior is lower and thus its precision is higher. When *N* = 600, model convergence results for BNP growth curve models follow the same pattern, and thus are not reported here.

For converged replications, we evaluate the impact of precision parameter priors on parameter estimation and computation time. Results for *N* = 200 are summarized in Tables 2–5. The relative performance of the four priors in conditions with a larger sample size (*N* = 600) has a similar pattern. Detailed results for *N* = 600 are available in the Supplementary Document.

**Table 2:** Model estimation for BNP growth curve modeling with different precision parameter priors when data are normal and *N* = 200.

**Prior**		**Est**.	**Bias**	**ASE**	**ESE**	**MSE**	**CP**	**AET**
*Gamma*(0.001, 0.001)	*β*_*L*_	6.204	0.004	0.082	0.084	0.007	0.957	539.332
	*β*_*S*_	0.301	0.001	0.033	0.032	0.001	0.957	539.332
	$\sigma_{L}^{2}$	0.999	–0.001	0.138	0.142	0.020	0.936	539.332
	$\sigma_{S}^{2}$	0.118	0.018	0.021	0.018	0.001	0.922	539.332
	*σ*_*LS*_	–0.010	–0.010	0.040	0.034	0.001	0.993	539.332
	$\sigma_{e}^{2}$	0.497	–0.003	0.024	0.036	0.001	0.816	539.332
	*K*	2.113	–	0.803	2.331	–	–	539.332
	*α*	11.134	–	18.109	104.805	–	–	539.332
*Gamma*(2, 2)	*β*_*L*_	6.198	–0.002	0.082	0.080	0.006	0.958	740.331
	*β*_*S*_	0.302	0.002	0.033	0.031	0.001	0.965	740.331
	$\sigma_{L}^{2}$	1.008	0.008	0.139	0.131	0.017	0.965	740.331
	$\sigma_{S}^{2}$	0.118	0.018	0.021	0.018	0.001	0.927	740.331
	*σ*_*LS*_	–0.010	–0.010	0.040	0.034	0.001	0.983	740.331
	$\sigma_{e}^{2}$	0.499	–0.001	0.024	0.034	0.001	0.823	740.331
	*K*	4.106	–	2.415	0.776	–	–	740.331
	*α*	0.732	–	0.526	0.126	–	–	740.331
*Gamma*(100, 100)	*β*_*L*_	6.200	0.000	0.083	0.083	0.007	0.948	1024.509
	*β*_*S*_	0.299	-0.001	0.033	0.032	0.001	0.958	1024.509
	$\sigma_{L}^{2}$	1.014	0.014	0.139	0.133	0.018	0.967	1024.509
	$\sigma_{S}^{2}$	0.117	0.017	0.021	0.018	0.001	0.942	1024.509
	*σ*_*LS*_	–0.010	–0.010	0.040	0.036	0.001	0.976	1024.509
	$\sigma_{e}^{2}$	0.499	–0.001	0.024	0.036	0.001	0.827	1024.509
	*K*	5.037	–	1.924	0.407	–	–	1024.509
	*α*	0.992	–	0.099	0.004	–	–	1024.509
*Gamma*(10, 100)	*β*_*L*_	6.202	0.002	0.082	0.082	0.007	0.945	370.307
	*β*_*S*_	0.301	0.001	0.033	0.031	0.001	0.971	370.307
	$\sigma_{L}^{2}$	1.001	0.001	0.138	0.129	0.017	0.971	370.307
	$\sigma_{S}^{2}$	0.117	0.017	0.021	0.018	0.001	0.942	370.307
	*σ*_*LS*_	–0.012	–0.012	0.040	0.037	0.001	0.974	370.307
	$\sigma_{e}^{2}$	0.498	–0.002	0.024	0.035	0.001	0.835	370.307
	*K*	1.981	–	0.874	0.199	–	–	370.307
	*α*	0.099	-	0.031	0.001	–	–	370.307

*Est, estimate; ASE, average standard error; ESE, empirical standard error; MSE, mean squared error; CP, coverage probability of the 95% HPD credible interval; AET, average estimation time*.

**Table 3:** Model estimation for BNP growth curve modeling with different precision parameter priors when data contain 5% of outliers and *N* = 200.

**Prior**		**Est**.	**Bias**	**ASE**	**ESE**	**MSE**	**CP**	**AET**
*Gamma*(0.001, 0.001)	*β*_*L*_	6.300	0.100	0.092	0.083	0.017	0.793	841.706
	*β*_*S*_	0.313	0.013	0.037	0.036	0.001	0.948	841.706
	$\sigma_{L}^{2}$	1.006	0.006	0.160	0.145	0.021	0.956	841.706
	$\sigma_{S}^{2}$	0.118	0.018	0.024	0.017	0.001	0.985	841.706
	*σ*_*LS*_	–0.009	–0.009	0.046	0.044	0.002	0.985	841.706
	$\sigma_{e}^{2}$	3.133	–	0.125	0.138	–	–	841.706
	*K*	5.184	–	1.189	5.433	–	–	841.706
	*α*	28.284	–	51.922	75.124	–	–	841.706
*Gamma*(2, 2)	*β*_*L*_	6.311	0.111	0.092	0.088	0.020	0.763	971.782
	*β*_*S*_	0.311	0.011	0.037	0.034	0.001	0.957	971.782
	$\sigma_{L}^{2}$	1.007	0.007	0.161	0.146	0.021	0.967	971.782
	$\sigma_{S}^{2}$	0.117	0.017	0.024	0.018	0.001	0.976	971.782
	*σ*_*LS*_	–0.008	–0.008	0.046	0.041	0.002	0.976	971.782
	$\sigma_{e}^{2}$	3.119	–	0.124	0.136	–	–	971.782
	*K*	6.515	–	2.905	0.981	- -	–	971.782
	*α*	1.126	–	0.676	0.171	–	–	971.782
*Gamma*(100, 100)	*β*_*L*_	6.298	0.098	0.091	0.090	0.018	0.794	1088.448
	*β*_*S*_	0.314	0.014	0.037	0.034	0.001	0.944	1088.448
	$\sigma_{L}^{2}$	0.987	–0.013	0.158	0.134	0.018	0.964	1088.448
	$\sigma_{S}^{2}$	0.117	0.017	0.024	0.018	0.001	0.976	1088.448
	*σ*_*LS*_	–0.004	–0.004	0.045	0.041	0.002	0.992	1088.448
	$\sigma_{e}^{2}$	3.133	–	0.124	0.130	–	–	1088.448
	*K*	6.161	–	1.930	0.467	–	–	1088.448
	*α*	1.003	–	0.100	0.005	–	–	1088.448
*Gamma*(10, 100)	*β*_*L*_	6.311	0.111	0.091	0.090	0.020	0.767	561.074
	*β*_*S*_	0.311	0.011	0.037	0.034	0.001	0.952	561.074
	$\sigma_{L}^{2}$	0.985	–0.015	0.158	0.144	0.021	0.960	561.074
	$\sigma_{S}^{2}$	0.119	0.019	0.024	0.018	0.001	0.964	561.074
	*σ*_*LS*_	–0.009	–0.009	0.046	0.042	0.002	0.968	561.074
	$\sigma_{e}^{2}$	3.118	–	0.124	0.136	–	–	561.074
	*K*	2.903	–	0.872	0.240	–	–	561.074
	*α*	0.103	–	0.032	0.001	–	–	561.074

*Est, estimate; ASE, average standard error; ESE, empirical standard error; MSE, mean squared error; CP, coverage probability of the 95% HPD credible interval; AET, average estimation time*.

**Table 4:** Model estimation for BNP growth curve modeling with different precision parameter priors when data contain 10% of outliers and *N* = 200.

**Prior**		**Est**.	**Bias**	**ASE**	**ESE**	**MSE**	**CP**	**AET**
*Gamma*(0.001, 0.001)	*β*_*L*_	6.437	0.237	0.103	0.102	0.066	0.348	591.282
	*β*_*S*_	0.335	0.035	0.043	0.039	0.003	0.917	591.282
	$\sigma_{L}^{2}$	1.018	0.018	0.187	0.173	0.030	0.977	591.282
	$\sigma_{S}^{2}$	0.126	0.026	0.028	0.022	0.001	0.917	591.282
	*σ*_*LS*_	–0.007	–0.007	0.053	0.047	0.002	0.977	591.282
	$\sigma_{e}^{2}$	5.464	–	0.173	0.180	–	–	591.282
	*K*	3.112	–	1.106	1.728	–	–	591.282
	*α*	1.041	–	2.885	7.422	–	–	591.282
*Gamma*(2, 2)	*β*_*L*_	6.424	0.224	0.103	0.105	0.061	0.426	938.496
	*β*_*S*_	0.336	0.036	0.043	0.038	0.003	0.886	938.496
	$\sigma_{L}^{2}$	1.020	0.020	0.187	0.174	0.031	0.966	938.496
	$\sigma_{S}^{2}$	0.121	0.021	0.027	0.020	0.001	0.970	938.496
	*σ*_*LS*_	–0.008	–0.008	0.053	0.044	0.002	0.979	938.496
	$\sigma_{e}^{2}$	5.448	–	0.172	0.171	–	–	938.496
	*K*	6.314	–	2.798	0.942	–	–	938.496
	*α*	1.090	–	0.652	0.163	–	–	938.496
*Gamma*(100, 100)	*β*_*L*_	6.428	0.228	0.104	0.100	0.062	0.398	1045.439
	*β*_*S*_	0.332	0.032	0.043	0.040	0.003	0.903	1045.439
	$\sigma_{L}^{2}$	1.020	0.020	0.188	0.172	0.030	0.964	1045.439
	$\sigma_{S}^{2}$	0.123	0.023	0.027	0.021	0.001	0.961	1045.439
	*σ*_*LS*_	–0.009	–0.009	0.053	0.043	0.002	0.982	1045.439
	$\sigma_{e}^{2}$	5.459	–	0.174	0.175	–	–	1045.439
	*K*	6.091	–	1.911	0.409	–	–	1045.439
	*α*	1.002	–	0.100	0.004	–	–	1045.439
*Gamma*(10, 100)	*β*_*L*_	6.426	0.226	0.103	0.102	0.062	0.395	389.282
	*β*_*S*_	0.333	0.033	0.043	0.041	0.003	0.897	389.282
	$\sigma_{L}^{2}$	1.011	0.011	0.185	0.177	0.032	0.957	389.282
	$\sigma_{S}^{2}$	0.123	0.023	0.027	0.021	0.001	0.943	389.282
	*σ*_*LS*_	–0.007	–0.007	0.052	0.045	0.002	0.975	389.282
	$\sigma_{e}^{2}$	5.457	–	0.172	0.169	–	–	389.282
	*K*	2.935	–	0.878	0.206	–	–	389.282
	*α*	0.103	–	0.032	0.001	–	–	389.282

*Est, estimate; ASE, average standard error; ESE, empirical standard error; MSE, mean squared error; CP, coverage probability of the 95% HPD credible interval; AET, average estimation time*.

**Table 5:** Model estimation for BNP growth curve modeling with different precision parameter priors when data contain 20% of outliers and *N* = 200.

**Prior**		**Est**.	**Bias**	**ASE**	**ESE**	**MSE**	**CP**	**AET**
*Gamma*(0.001, 0.001)	*β*_*L*_	6.890	0.690	0.149	0.120	0.490	0.000	460.170
	*β*_*S*_	0.385	0.085	0.061	0.054	0.010	0.735	460.170
	$\sigma_{L}^{2}$	1.321	0.321	0.315	0.284	0.183	0.884	460.170
	$\sigma_{S}^{2}$	0.141	0.041	0.038	0.027	0.002	0.952	460.170
	*σ*_*LS*_	0.019	0.019	0.080	0.062	0.004	0.980	460.170
	$\sigma_{e}^{2}$	9.238	–	0.242	0.258	–	–	460.170
	*K*	2.713	–	0.810	0.307	–	–	460.170
	*α*	0.019	–	0.047	0.089	–	–	460.170
*Gamma*(2, 2)	*β*_*L*_	6.890	0.690	0.150	0.120	0.490	0.000	949.186
	*β*_*S*_	0.381	0.081	0.061	0.052	0.009	0.787	949.186
	$\sigma_{L}^{2}$	1.358	0.358	0.321	0.279	0.206	0.879	949.186
	$\sigma_{S}^{2}$	0.143	0.043	0.038	0.024	0.002	0.962	949.186
	*σ*_*LS*_	0.011	0.011	0.082	0.064	0.004	0.983	949.186
	$\sigma_{e}^{2}$	9.167	–	0.245	0.265	–	–	949.186
	*K*	5.458	–	2.392	0.564	–	–	949.186
	*α*	0.941	–	0.566	0.095	–	–	949.186
*Gamma*(100, 100)	*β*_*L*_	6.882	0.682	0.149	0.121	0.480	0.000	1056.953
	*β*_*S*_	0.381	0.081	0.061	0.054	0.010	0.774	1056.953
	$\sigma_{L}^{2}$	1.323	0.323	0.314	0.284	0.185	0.878	1056.953
	$\sigma_{S}^{2}$	0.143	0.043	0.038	0.026	0.003	0.944	1056.953
	*σ*_*LS*_	0.010	0.010	0.081	0.062	0.004	0.981	1056.953
	$\sigma_{e}^{2}$	9.172	–	0.243	0.256	–	–	1056.953
	*K*	5.695	–	1.811	0.321	–	–	1056.953
	*α*	0.998	–	0.099	0.003	–	–	1056.953
*Gamma*(10, 100)	*β*_*L*_	6.897	0.697	0.150	0.116	0.499	0.000	391.429
	*β*_*S*_	0.379	0.079	0.061	0.052	0.009	0.803	391.429
	$\sigma_{L}^{2}$	1.354	0.354	0.319	0.280	0.204	0.861	391.429
	$\sigma_{S}^{2}$	0.141	0.041	0.038	0.026	0.002	0.956	391.429
	*σ*_*LS*_	0.014	0.014	0.081	0.064	0.004	0.980	391.429
	$\sigma_{e}^{2}$	9.166	–	0.242	0.255	–	–	391.429
	*K*	2.880	–	0.855	0.151	–	–	391.429
	*α*	0.103	-	0.032	0.001	–	–	391.429

*Est, estimate; ASE, average standard error; ESE, empirical standard error; MSE, mean squared error; CP, coverage probability of the 95% HPD credible interval; AET, average estimation time*.

From Tables 2–5, we obtain the following findings. First, the estimates of growth curve parameters ($\beta_{L},\beta_{S},\sigma_{L}^{2},\sigma_{S}^{2},\sigma_{LS},\sigma_{e}^{2}$) are not affected by different priors. Estimation bias, standard errors, MSE, and coverage probability of the 95% HPD credible interval across different precision parameter prior conditions are very close to each other, respectively. Note that when outliers exist (see Tables 3–5), the true population parameter value of the measurement error variance $\sigma_{e}^{2}$ is unknown. So, bias, MSE, and CP for this parameter cannot be calculated.

Second, the estimation of the hyperparameter *α* is greatly affected by different priors. When the non-informative prior is used, the estimated *α* can be very large (e.g., 28.284 in Table 3) or small (e.g., 0.019 in Table 5), associating with a large standard error. When *Gamma*(2, 2) or *Gamma*(100, 100) is used, estimated *α* is almost always close to 1. When *Gamma*(10, 100) is used, estimated *α* is around 0.1. Different *α* values indicate a different total number of classes *K*. In general, a larger *α* value may yield a larger number of latent classes. Since the estimated *α* has a large standard error when the non-informative diffuse prior is used, the corresponding estimated *K* can also be large or small. For the weakly informative and accurate informative priors, the estimated number of latent classes ranges from 4 to 6 for different data conditions, whereas for the inaccurate informative prior, the estimated number of latent classes is about 2 or 3. It is interesting to see that although distinctively different hyperparameter estimates are obtained leading to different number of latent classes, the estimated growth curve parameters are essentially similar. This is because although outliers are generated from 10 different distributions, the 10 different distributions are not separated far apart. With a low class separation, one distribution may be enough to describe several outliers generated from different distributions. Thus, even the inaccurate informative prior can yield a precision parameter that is adequate to model the measurement errors.

Third, BNP growth curve modeling with the inaccurate informative prior *Gamma*(10, 100) requires the shortest computation time. This is because the inaccurate informative prior here has the smallest variance and thus is most “informative” among the four priors.

Fourth, outliers affect the performance of BNP growth curve modeling. When data contain a large proportion of outliers (e.g., 20%), estimation bias for the average of random intercepts *β*_*L*_ and variance of random intercepts $\sigma_{L}^{2}$ are much larger than those when outlier proportion is low. In addition, outliers influence computation time. It is worth mentioning that it is most time consuming when the outlier proportion is 5%. A possible reason is that a small proportion of outliers creates a steep and high-curvature region for Markov chains to enter and thus takes longer time to converge. With more outliers, the curvature is smoother so the computation is faster.

## 5. Discussion

Restricting to a parametric probability family can delude investigators and falsely make an illusion of posterior certainty (Müller and Mitra, 2004). On the contrary, BNP methods are adaptive and powerful to discover complex patterns in real data. Although BNP growth curve modeling has been proposed, the effect of the precision parameter was not fully studied. In this article, we have conducted a simulation study to investigate how different types of precision parameter priors impact the convergence rate, model estimation, and computation time in BNP growth curve modeling. We found that the non-informative prior suffered from the lowest convergence rates while the inaccurate informative prior with the smallest prior variance yielded the highest convergence rates and the fastest computations. Furthermore, we found that the estimation of growth curve parameters was not affected by the prior of the precision parameter. Based on these results, we recommend to use informative priors with high precision in practice.

We would like to note that although it seems counterintuitive that the inaccurate informative prior for the precision parameter performed the best, such findings have been observed in the literature. For example, Finch and Miller (2019) found that slightly informative priors can be advantageous in small samples even when these priors are incorrect. Depaoli (2013) showed that growth mixture model estimations obtained with inaccurate priors were still more accurate than maximum likelihood or Bayesian estimation with diffuse priors. Zitzmann et al. (2020) explicitly discussed this issue for small samples. Our simulation results also supported the argument that the amount of information in the prior can be more important than the accuracy of the prior under certain circumstances.

We also want to point out that the estimation bias was relatively large in our simulation study, when compared to that in previous studies (Tong and Zhang, 2019). This is because we consider much higher outlier proportions. When the outlier proportion is low (i.e., 5%), parameter estimates are very close to the true population values. As the outlier proportion increases, the bias increases. One possible way to improve the performance of BNP growth curve modeling when the outlier proportion is high is to use a non-normal base distribution. In our simulation study, for simplicity, we used normal distributions with zero mean as the mixing components of BNP modeling. This cannot handle asymmetric non-normal distributions, which may partly explain the less satisfactory performance of BNP modeling in the conditions with high outlier proportions. But BNP methods in general are very flexible. A non-normal base distribution may overcome this limitation. While future studies may continue along this path, we want to emphasize that BNP modeling as in our study still outperforms traditional growth curve modeling and is recommended to use in general when data are suspected to be non-normal (Tong and Zhang, 2019) no matter the non-normality is caused by non-normal population distribution or data contamination.

The convergence rate of BNP growth curve modeling was found to be higher in previous studies, i.e., close to one (Tong and Zhang, 2019). We would like to note that the difference is likely due to the list of parameters counted during convergence assessment. In Tong and Zhang (2019), the convergence rate was computed only for growth curve parameters. When only growth curve parameters are considered, non-convergence rarely occurred in our study. The major problem is the precision parameter. As shown in the simulation study, non-convergence frequently arose for this parameter (detailed Geweke tests results for each parameter are available on our GitHub site: https://github.com/CynthiaXinTong/PrecisionParPrior_BNP_GCM). Another possible reason why convergence rates were relatively low (below 70%) in our simulation is that Geweke tests often yield lower rates of convergence than other diagnostic methods (e.g., Jang and Cohen, 2020). However, as pointed out in Jang and Cohen, the pattern of convergence rates for model comparison was similar for different diagnostic tests. Namely, our conclusions about which precision parameter priors to use in BNP growth curve modeling will not be affected by the diagnostic tests. We further discuss the use of Geweke tests in the next paragraph. Notably, although the non-convergence for the precision parameter seemed not to impact parameter estimates for the growth curve parameters, such issue may mislead model fit assessment. Although model assessment and model comparison methods have been proposed for various models, samples of different sizes, and data structures (e.g., Celeux et al., 2006), their performance in BNP analysis has not been studied. Therefore, future studies on how different precision parameter priors affect model fit assessment are encouraged.

In our study, model convergence diagnostics were conducted using Geweke tests. Although Geweke tests are commonly used in the Bayesian literature, it is impossible to say with certainty that a finite sample from an MCMC algorithm is representative of an underlying stationary distribution and a combination of strategies aiming at evaluating and accelerating MCMC sampler convergence is recommended (Cowles and Carlin, 1996). For our simulation study, Geweke tests were relatively easy to systematically implement. In empirical studies, we recommend using multiple strategies (e.g., trace plots, multiple chains) to check model convergence. In addition, since Zitzmann and Hecht (2019) pointed out that it is possible that the approximation of the Bayesian estimates is still not optimal even when a chain converges, we recommend substantive researchers conducting sensitivity analysis and evaluating how the length of the Markov chains affects the model estimation results.

Our study echoed the previous literature in that using informative priors may help reduce computation time in Bayesian modeling. We would like to note that there are other approaches that can be used to further increase the computation efficiency. For example, Berger et al. (2020) and Daniels and Kass (1999) proposed shrinkage priors, and Hecht et al. (2020) proposed a model reformulation approach in which the sample covariance matrix was modeled instead of individual observations. This latter approach has been applied to the Bayesian continuous-time model (Hecht and Zitzmann, 2020) as well as the Bayesian STARTS model (Ludtke et al., 2018). Future research on BNP growth curve modeling could incorporate this approach and other potentially efficient approaches to reduce computation time.

The employment of BNP growth curve modeling is a field still in its early stage. New DP variants and generalizations are being proposed every year to cater to specific applications. BNP modeling was only used to handle the non-normality in intraindividual measurement errors in our study. The similar strategy can be used for random effects, such as random intercepts and slopes. Also, although we worked with balanced data, BNP growth curve modeling should be able to handle unbalanced data (e.g., individually varying time points). However, as implied by previous studies (Tong, 2014), the convergence issue may be more challenging, thereby awaiting future studies.

## Data Availability Statement

The raw data supporting the conclusions of this article are available by running the data generation R code on our GitHub site: https://github.com/CynthiaXinTong/PrecisionParPrior_BNP_GCM.

## Author Contributions

All authors listed have made a substantial, direct and intellectual contribution to the work, and approved it for publication.

## Conflict of Interest

The authors declare that the research was conducted in the absence of any commercial or financial relationships that could be construed as a potential conflict of interest.

## Footnotes

^1^In practice, infinite-dimension means finite but unbounded dimension.

^2^Note that non-normal data may be caused by non-normal population distributions or data contaminations. We work with outliers in this simulation study because BNP methods are essentially infinite mixture modeling procedures. Generating and dealing with outliers from multiple different distributions are more manageable as we easily know the true number of underlying classes. It is worth verifying the conclusions of this paper for non-normal population distributions in the future.

^3^Multiple lengths of Markov chains were tested before the current setting was selected. The convergence results with 50,000 iterations were about the same as those for longer chains.

^4^ASE is the mean estimated standard error across replications. ESE is the standard deviation of the parameter estimates from all replications. MSE is computed as squared bias plus squared ESE. Posterior credible interval, also called credible interval, is the Bayesian counterpart of the frequentist confidence interval. A HPD interval is essentially the narrowest interval on a posterior that covers a given proportion of the probable posterior values.
