# Supplementary material for: Assessing the Impact of Precision Parameter Prior in Bayesian Non-parametric Growth Curve Modeling
Source: Front Psychol. 2021 Mar 31;12:624588. doi: 10.3389/fpsyg.2021.624588 (PMC8044365; doi:10.3389/fpsyg.2021.624588)
Supplement: Supplementary file 1 [file Data_Sheet_1.pdf]

Table 1: Model estimation for BNP growth curve modeling with different precision parameter priors when data are normal and $N = 600$

Prior		Est.	Bias	ASE	ESE	MSE	CP	AET
$Gamma(0.001, 0.001)$	β_L	6.200	0.000	0.048	0.044	0.002	0.965	1440.371
	β_S	0.299	-0.001	0.018	0.018	0.000	0.942	1440.371
	σ_L^2	1.009	0.009	0.080	0.080	0.006	0.954	1440.371
	σ_S^2	0.104	0.004	0.012	0.012	0.000	0.913	1440.371
	σ_{LS}	-0.005	-0.005	0.023	0.021	0.000	0.960	1440.371
	σ_e^2	0.499	-0.001	0.014	0.019	0.000	0.850	1440.371
	K	1.955	-	0.794	0.198	-	-	1440.371
	α	0.000	-	0.001	0.002	-	-	1440.371
$Gamma(2, 2)$	β_L	6.198	-0.002	0.048	0.046	0.002	0.947	2232.016
	β_S	0.298	-0.002	0.019	0.019	0.000	0.955	2232.016
	σ_L^2	1.007	0.007	0.080	0.078	0.006	0.955	2232.016
	σ_S^2	0.105	0.005	0.012	0.011	0.000	0.947	2232.016
	σ_{LS}	-0.004	-0.004	0.023	0.023	0.001	0.955	2232.016
	σ_e^2	0.499	-0.001	0.014	0.021	0.000	0.818	2232.016
	K	4.042	-	2.316	0.850	-	-	2232.016
	α	0.612	-	0.430	0.116	-	-	2232.016
$Gamma(100, 100)$	β_L	6.197	-0.003	0.048	0.045	0.002	0.969	3207.358
	β_S	0.300	0.000	0.018	0.019	0.000	0.918	3207.358
	σ_L^2	1.010	0.010	0.080	0.080	0.006	0.949	3207.358
	σ_S^2	0.104	0.004	0.012	0.011	0.000	0.953	3207.358
	σ_{LS}	-0.003	-0.003	0.023	0.022	0.000	0.965	3207.358
	σ_e^2	0.499	-0.001	0.014	0.020	0.000	0.855	3207.358
	K	5.572	-	2.076	0.482	-	-	3207.358
	α	0.987	-	0.099	0.005	-	-	3207.358
$Gamma(10, 100)$	β_L	6.196	-0.004	0.048	0.043	0.002	0.977	1491.113
	β_S	0.301	0.001	0.019	0.019	0.000	0.934	1491.113
	σ_L^2	1.007	0.007	0.080	0.080	0.006	0.948	1491.113
	σ_S^2	0.106	0.006	0.012	0.012	0.000	0.928	1491.113
	σ_{LS}	-0.004	-0.004	0.023	0.022	0.001	0.944	1491.113
	σ_e^2	0.498	-0.002	0.014	0.019	0.000	0.856	1491.113
	K	2.157	-	0.904	0.212	-	-	1491.113
	α	0.099	-	0.031	0.001	-	-	1491.113

Note. Est. = estimate; ASE = average standard error; ESE = empirical standard error; MSE = mean squared error; CP = coverage probability of the 95% HPD credible interval; AET = average estimation time.

Table 2: Model estimation for BNP growth curve modeling with different precision parameter priors when data contain 5% of outliers and $N = 600$

Prior		Est.	Bias	ASE	ESE	MSE	CP	AET
$Gamma(0.001, 0.001)$	β_L	6.295	0.095	0.053	0.048	0.011	0.535	2632.864
	β_S	0.316	0.016	0.021	0.018	0.001	0.916	2632.864
	σ_L^2	0.993	-0.007	0.091	0.080	0.006	0.955	2632.864
	σ_S^2	0.106	0.006	0.014	0.013	0.000	0.961	2632.864
	σ_{LS}	-0.002	-0.002	0.026	0.027	0.001	0.974	2632.864
	σ_e^2	3.121	-	0.072	0.075	-	-	2632.864
	K	4.123	-	0.907	4.300	-	-	2632.864
	α	15.678	-	24.329	62.946	-	-	2632.864
$Gamma(2, 2)$	β_L	6.296	0.096	0.053	0.048	0.012	0.553	3107.725
	β_S	0.314	0.014	0.021	0.020	0.001	0.916	3107.725
	σ_L^2	0.986	-0.014	0.091	0.086	0.008	0.942	3107.725
	σ_S^2	0.105	0.005	0.014	0.013	0.000	0.953	3107.725
	σ_{LS}	0.000	0.000	0.026	0.025	0.001	0.963	3107.725
	σ_e^2	3.139	-	0.072	0.075	-	-	3107.725
	K	6.614	-	2.977	1.276	-	-	3107.725
	α	0.964	-	0.575	0.187	-	-	3107.725
$Gamma(100, 100)$	β_L	6.304	0.104	0.052	0.052	0.013	0.492	3448.362
	β_S	0.316	0.016	0.021	0.023	0.001	0.862	3448.362
	σ_L^2	0.981	-0.019	0.090	0.080	0.007	0.949	3448.362
	σ_S^2	0.106	0.006	0.014	0.012	0.000	0.964	3448.362
	σ_{LS}	0.000	0.000	0.026	0.027	0.001	0.944	3448.362
	σ_e^2	3.126	-	0.072	0.076	-	-	3448.362
	K	6.870	-	2.107	0.528	-	-	3448.362
	α	0.999	-	0.100	0.005	-	-	3448.362
$Gamma(10, 100)$	β_L	6.296	0.096	0.053	0.051	0.012	0.569	1859.082
	β_S	0.314	0.014	0.021	0.022	0.001	0.881	1859.082
	σ_L^2	0.991	-0.009	0.091	0.090	0.008	0.942	1859.082
	σ_S^2	0.106	0.006	0.014	0.013	0.000	0.958	1859.082
	σ_{LS}	-0.001	-0.001	0.026	0.025	0.001	0.950	1859.082
	σ_e^2	3.134	-	0.072	0.074	-	-	1859.082
	K	3.116	-	0.922	0.296	-	-	1859.082
	α	0.103	-	0.032	0.001	-	-	1859.082

Note. Est. = estimate; ASE = average standard error; ESE = empirical standard error; MSE = mean squared error; CP = coverage probability of the 95% HPD credible interval; AET = average estimation time.

Table 3: Model estimation for BNP growth curve modeling with different precision parameter priors when data contain 10% of outliers and $N = 600$

Prior		Est.	Bias	ASE	ESE	MSE	CP	AET
$Gamma(0.001, 0.001)$	β_L	6.433	0.233	0.060	0.051	0.057	0.000	1507.879
	β_S	0.330	0.030	0.024	0.024	0.001	0.750	1507.879
	σ_L^2	1.023	0.023	0.107	0.102	0.011	0.949	1507.879
	σ_S^2	0.109	0.009	0.016	0.015	0.000	0.942	1507.879
	σ_{LS}	0.002	0.002	0.030	0.028	0.001	0.981	1507.879
	σ_e^2	5.459	-	0.100	0.109	-	-	1507.879
	K	3.076	-	1.026	0.890	-	-	1507.879
	α	0.078	-	0.167	0.447	-	-	1507.879
$Gamma(2, 2)$	β_L	6.427	0.227	0.060	0.049	0.054	0.009	2769.165
	β_S	0.330	0.030	0.024	0.023	0.001	0.771	2769.165
	σ_L^2	1.027	0.027	0.108	0.098	0.010	0.963	2769.165
	σ_S^2	0.108	0.008	0.016	0.014	0.000	0.967	2769.165
	σ_{LS}	0.001	0.001	0.030	0.028	0.001	0.986	2769.165
	σ_e^2	5.465	-	0.100	0.109	-	-	2769.165
	K	6.637	-	2.952	1.172	-	-	2769.165
	α	0.966	-	0.571	0.171	-	-	2769.165
$Gamma(100, 100)$	β_L	6.432	0.232	0.060	0.057	0.057	0.032	3263.632
	β_S	0.328	0.028	0.024	0.021	0.001	0.833	3263.632
	σ_L^2	1.017	0.017	0.107	0.094	0.009	0.977	3263.632
	σ_S^2	0.108	0.008	0.016	0.014	0.000	0.955	3263.632
	σ_{LS}	0.001	0.001	0.030	0.027	0.001	0.991	3263.632
	σ_e^2	5.466	-	0.100	0.104	-	-	3263.632
	K	6.850	-	2.104	0.525	-	-	3263.632
	α	0.999	-	0.099	0.005	-	-	3263.632
$Gamma(10, 100)$	β_L	6.429	0.229	0.060	0.058	0.056	0.031	1693.155
	β_S	0.331	0.031	0.024	0.023	0.001	0.771	1693.155
	σ_L^2	1.017	0.017	0.107	0.103	0.011	0.958	1693.155
	σ_S^2	0.109	0.009	0.016	0.016	0.000	0.931	1693.155
	σ_{LS}	0.003	0.003	0.030	0.026	0.001	0.989	1693.155
	σ_e^2	5.461	-	0.100	0.112	-	-	1693.155
	K	3.205	-	0.941	0.285	-	-	1693.155
	α	0.103	-	0.032	0.001	-	-	1693.155

Note. Est. = estimate; ASE = average standard error; ESE = empirical standard error; MSE = mean squared error; CP = coverage probability of the 95% HPD credible interval; AET = average estimation time.

Table 4: Model estimation for BNP growth curve modeling with different precision parameter priors when data contain 20% of outliers and $N = 600$

Prior		Est.	Bias	ASE	ESE	MSE	CP	AET
$Gamma(0.001, 0.001)$	β_L	6.893	0.693	0.087	0.068	0.485	0.000	1618.458
	β_S	0.373	0.073	0.034	0.031	0.006	0.435	1618.458
	σ_L^2	1.378	0.378	0.185	0.173	0.173	0.468	1618.458
	σ_S^2	0.114	0.014	0.021	0.017	0.000	0.968	1618.458
	σ_{LS}	0.029	0.029	0.046	0.042	0.003	0.883	1618.458
	σ_e^2	9.172	-	0.140	0.142	-	-	1618.458
	K	2.872	-	0.803	0.222	-	-	1618.458
	α	0.005	-	0.021	0.015	-	-	1618.458
$Gamma(2, 2)$	β_L	6.887	0.687	0.087	0.069	0.477	0.000	2931.519
	β_S	0.376	0.076	0.034	0.031	0.007	0.384	2931.519
	σ_L^2	1.362	0.362	0.184	0.167	0.159	0.507	2931.519
	σ_S^2	0.113	0.013	0.021	0.016	0.000	0.956	2931.519
	σ_{LS}	0.031	0.031	0.046	0.040	0.003	0.904	2931.519
	σ_e^2	9.185	-	0.140	0.147	-	-	2931.519
	K	5.853	-	2.642	0.736	-	-	2931.519
	α	0.852	-	0.513	0.105	-	-	2931.519
$Gamma(100, 100)$	β_L	6.884	0.684	0.087	0.067	0.473	0.000	3333.843
	β_S	0.374	0.074	0.034	0.032	0.007	0.410	3333.843
	σ_L^2	1.357	0.357	0.184	0.169	0.156	0.486	3333.843
	σ_S^2	0.113	0.013	0.021	0.017	0.000	0.964	3333.843
	σ_{LS}	0.027	0.027	0.046	0.038	0.002	0.920	3333.843
	σ_e^2	9.180	-	0.141	0.146	-	-	3333.843
	K	6.435	-	2.030	0.409	-	-	3333.843
	α	0.995	-	0.099	0.004	-	-	3333.843
$Gamma(10, 100)$	β_L	6.893	0.693	0.087	0.065	0.484	0.000	1471.815
	β_S	0.373	0.073	0.034	0.030	0.006	0.403	1471.815
	σ_L^2	1.377	0.377	0.185	0.180	0.174	0.485	1471.815
	σ_S^2	0.114	0.014	0.021	0.017	0.000	0.947	1471.815
	σ_{LS}	0.029	0.029	0.046	0.042	0.003	0.904	1471.815
	σ_e^2	9.170	-	0.140	0.147	-	-	1471.815
	K	3.078	-	0.911	0.200	-	-	1471.815
	α	0.103	-	0.032	0.001	-	-	1471.815

Note. Est. = estimate; ASE = average standard error; ESE = empirical standard error; MSE = mean squared error; CP = coverage probability of the 95% HPD credible interval; AET = average estimation time.

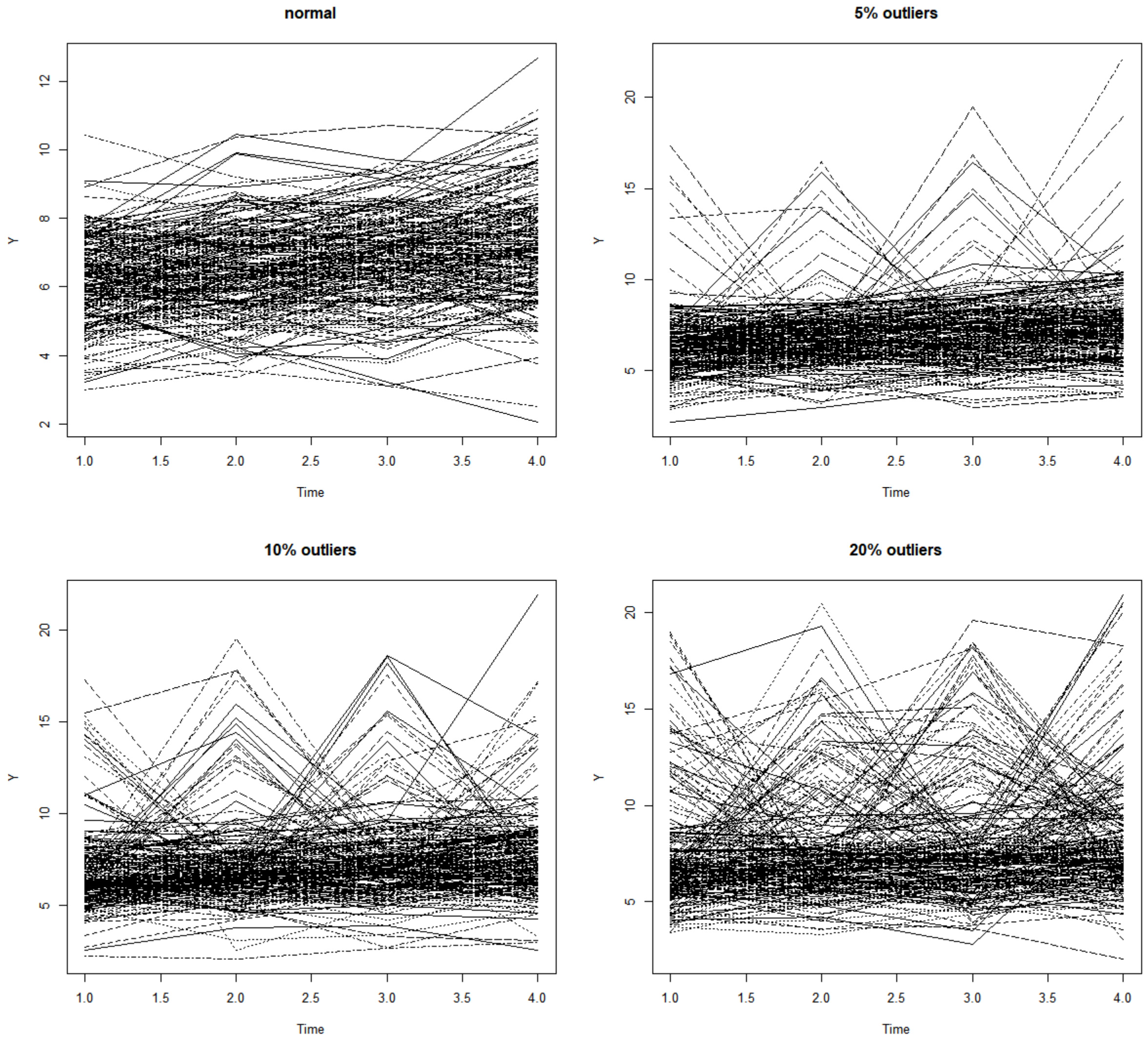

Figure 1: Trajectory plots of a set of generated normal data and corresponding data with outliers when $N = 200$

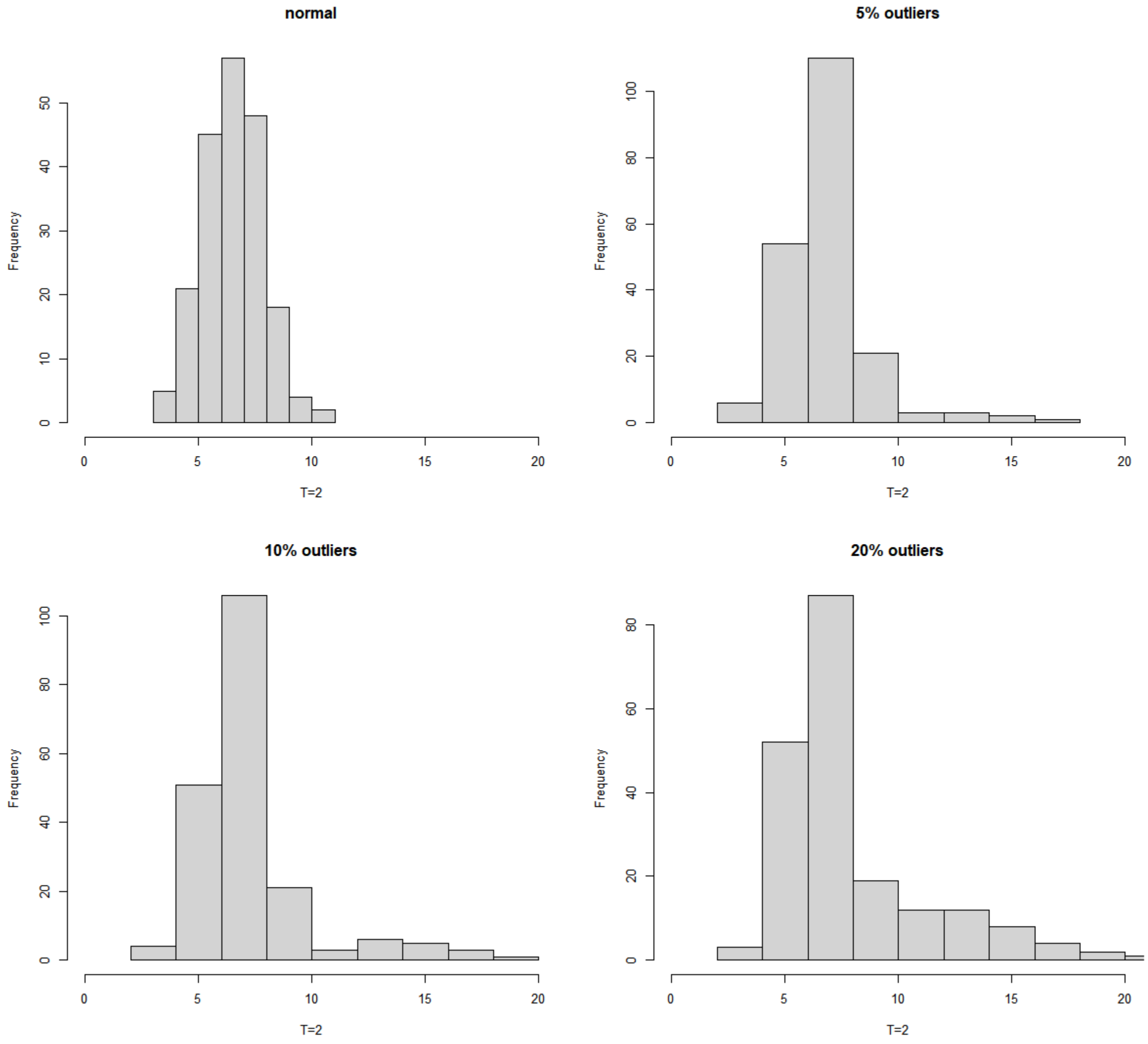

Figure 2: Histograms of a set of generated normal data and corresponding data with outliers at $T = 2$ when $N = 200$

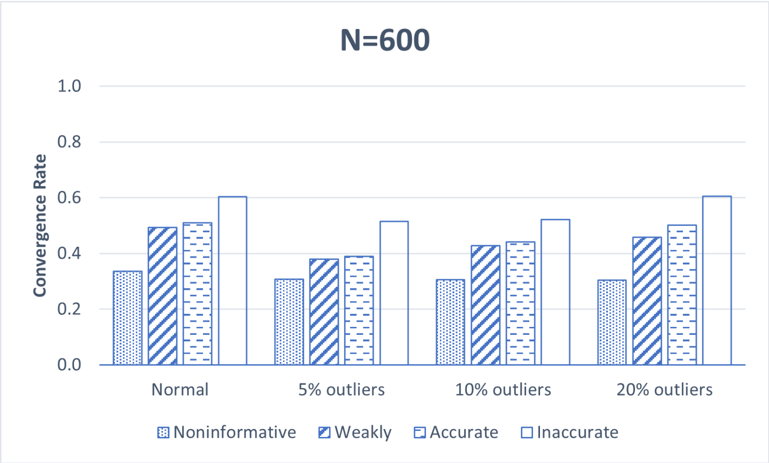

Figure 3: Convergence rate for different priors when $N = 600$

Appendix. Implementation

To facilitate the application of BNP growth curve modeling, we illustrate how it can be implemented in free software R using the rjags package. First, we specify the model using JAGS code and save it into a text file model.txt.

```
model{
  # Model specification for BNP linear growth curve model
        for (i in 1:N){
                LS[i,1:2]~dmnorm(muLS[i,1:2], Inv_cov[1:2,1:2])

                muLS[i,1]<-bL[1]
        muLS[i,2]<-bS[1]

                for (t in 1:T){
                        y[i, t] ~ dnorm(muY[i,t], taue[i])
                        muY[i,t] <- LS[i,1]+LS[i,2]*(t-1)
                }
                taue[i] <- taue.mix[groupe[i]]
                groupe[i] ~ dcat(pei[])

                for (j in 1:C){ ##C is the largest possible #number of classes
                   , can be set at a large number, e.g., 20.
                        ginde[i,j] <- equals(j,groupe[i])
                }
        }

        #Priors for model parameter
        for (i in 1:1){
        bL[i] ~ dnorm(0, 1.0E-6)
                bS[i] ~ dnorm(0, 1.0E-6)
        }

        ## truncated stick breaking construction
        pe[1]<-qe[1]
        for (j in 2:C){
                pe[j] <- qe[j] * (1 - qe[j - 1]) * pe[j -1 ] / qe[j - 1]
        }

        for (j in 1:C){
                qe[j] ~dbeta(1, alpha)T(0.0001,0.9999)
                pei[j] <- pe[j]/sum(pe[])
                taue.mix[j] ~ dgamma(aprece,bprece)
        }

        ##DP precision parameter,4 different priors were used in #our
```

```
   simulation study
alpha~dgamma(100,100)

aprece <- 2
bprece ~dgamma(2,2)

##total clusters
Ke <- sum(cle[])
for (j in 1:20) {
        suminde[j] <- sum(ginde[,j])
        cle[j] <- step(suminde[j]-1)
}

Inv_cov[1:2,1:2]~dwish(R[1:2,1:2], 2)
R[1,1]<-1
R[2,2]<-1
R[2,1]<-R[1,2]
R[1,2]<-0

para[1] <- bL[1]
para[2] <- bS[1]

Cov[1:2,1:2]<-inverse(Inv_cov[1:2,1:2])
para[3] <- Cov[1,1]
para[4] <- Cov[2,2]
para[5] <- Cov[1,2]


for (i in 1:N){
        for (t in 1:T){
                par[i,t] <- y[i,t]-LS[i,1]-LS[i,2]*(t-1)
        }
}

for (t in 1:T){
        for(i in 1:N){
        err[(t-1)*N+i] <- par[i,t]
        }
}

para[6] <- sd(err[])*sd(err[])
para[7] <- Ke
para[8] <- alpha
```

```
        para[9] <- bprece
}
```

JAGS has been integrated with the R software environment. To run the above JAGS code in R, we first install and load the rjags package.

```
install.packages("rjags")
library(rjags)
```

Then, we prepare the data, the initial values, and run jags.

```
##prepare data
data <- read.table('data.txt')
N <- nrow(data)
jagsdata <- list(N=N, T=4, C=20, y=as.matrix(data))

##specify initial values
inits <- list(Inv_cov = structure(.Data = c(1.0,0.0,0.0,10.0), .Dim = c(2,2)),
  alpha = 1.0, bL = c(6.2),bS = c(0.3),bprece = 0.5,".RNG.name" = "base::
     Wichmann-Hill", ".RNG.seed" = 115)
#note that  we specified the random number generator and the seed
#so our study can be replicated.

##run jags
#save the start time
time0 <- proc.time()

#read the model, burn 25,000 iterations
model <- jags.model(file="model.txt", data=jagsdata, inits=inits, n.chains =
    1, n.adapt=25000)

#run 25,000 iterations after the burn-in priord
model.samples <- coda.samples(model, c("para"), n.iter=25000)

#save results into model.res
model.res <- as.mcmc(do.call(rbind,model.samples))

#obtain the estimation time: end time - start time
time1 <- proc.time()-time0
```

Finally, we extract the model estimation results from model.res.

```
#parameter estimates
summary(model.res)

#HPD credible intervals
```

```
HPDinterval(model.res)

#geweke tests
geweke.diag(model.res)
```
