# Supplementary figures and images for: Assessing the Impact of Precision Parameter Prior in Bayesian Non-parametric Growth Curve Modeling
Source: Front Psychol. 2021 Mar 31;12:624588. doi: 10.3389/fpsyg.2021.624588 (PMC8044365; doi:10.3389/fpsyg.2021.624588)

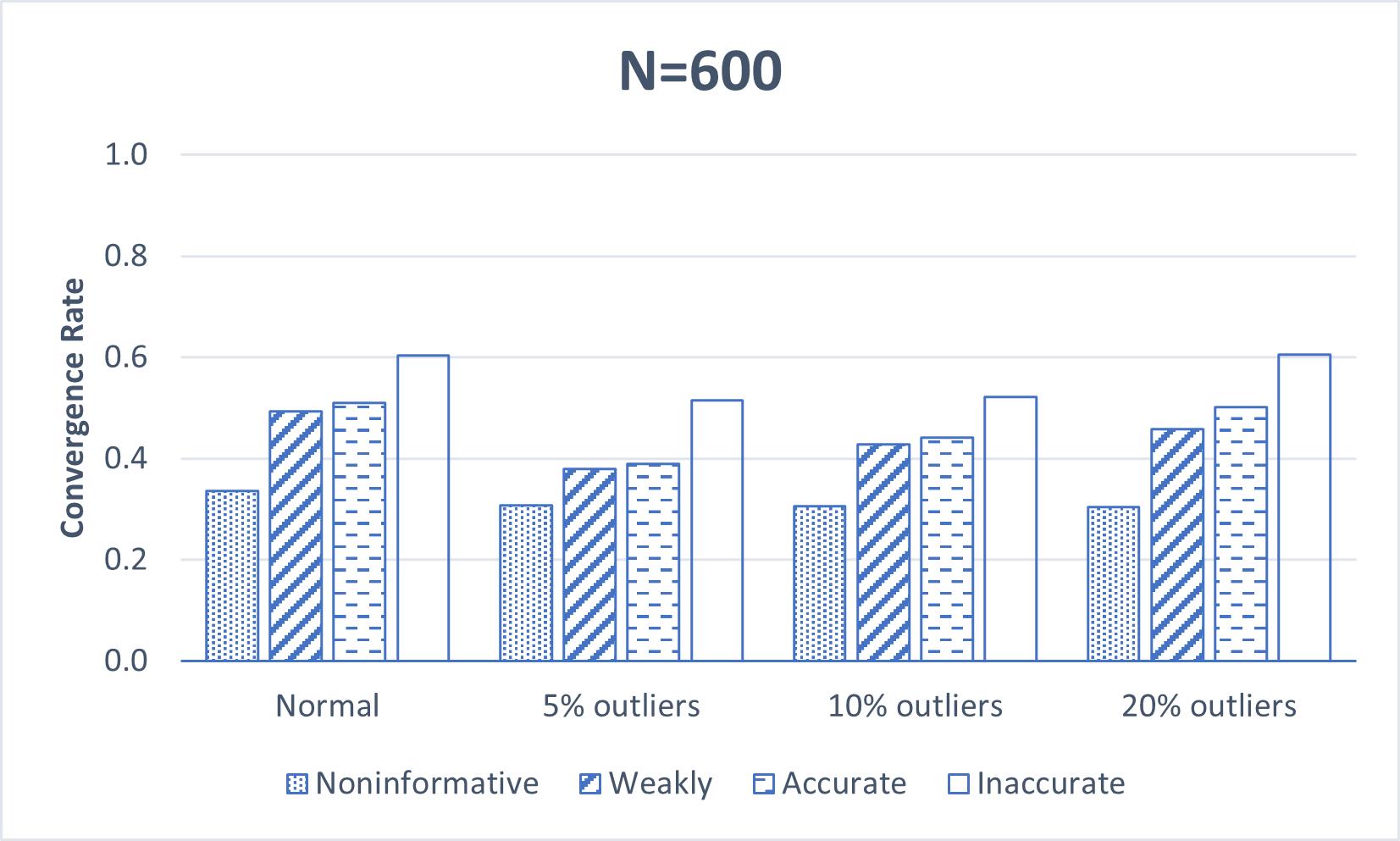

Supplement: Supplementary file 2 [file Image_1.JPEG]

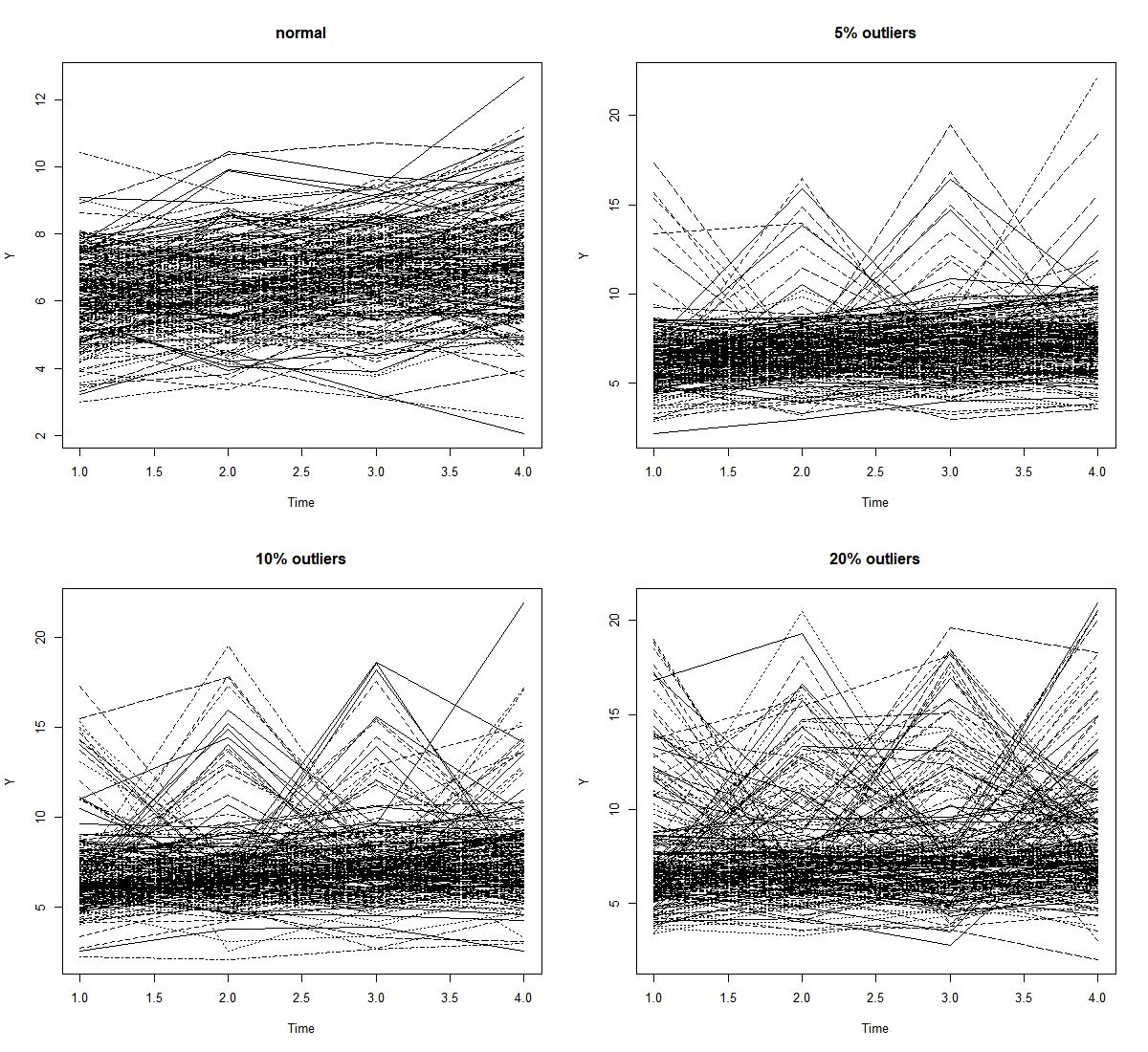

Supplement: Supplementary file 3 [file Image_2.JPEG]

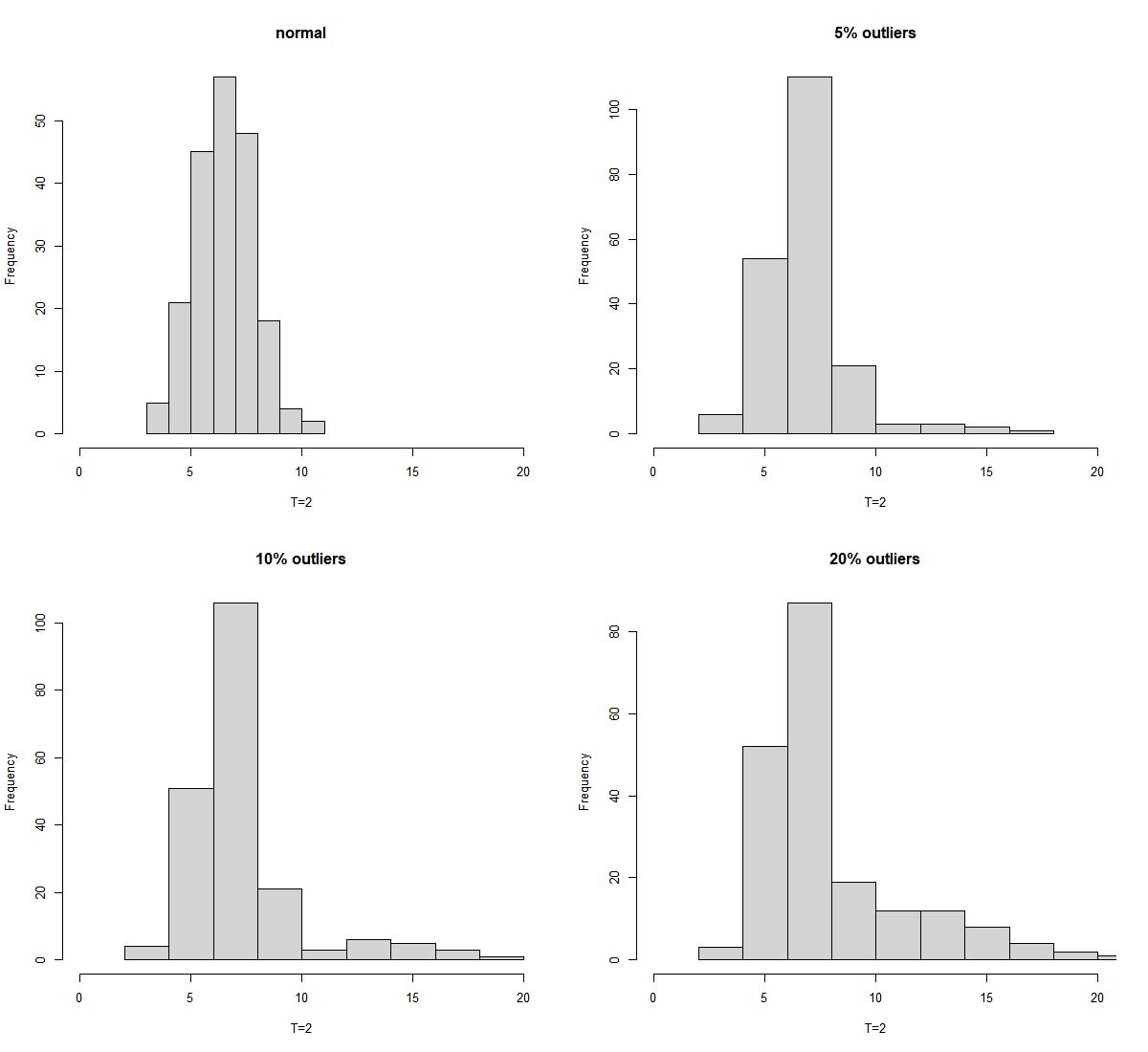

Supplement: Supplementary file 4 [file Image_3.JPEG]
